# Caring contact SMS text messages following suicidal behaviour

**DOI:** 10.1177/10398562251382455

**Published:** 2025-10-07

**Authors:** Lillian Ng, Danielle Diamond, Denisse Sanchez, Mike Ang

**Affiliations:** Department of Psychological Medicine, Faculty of Medical and Health Sciences, 1415 The University of Auckland, Auckland, New Zealand; Division of Mental Health & Addictions, 10158 Health New Zealand Te Whatu Ora Counties Manukau, Auckland, New Zealand; Division of Mental Health & Addictions, 10158 Health New Zealand Te Whatu Ora Counties Manukau, Auckland, New Zealand; Department of Psychological Medicine, Faculty of Medical and Health Sciences, 1415 The University of Auckland, Auckland, New Zealand; Division of Mental Health & Addictions, 10158 Health New Zealand Te Whatu Ora Counties Manukau, Auckland, New Zealand; Division of Mental Health & Addictions, 10158 Health New Zealand Te Whatu Ora Counties Manukau, Auckland, New Zealand

**Keywords:** psychiatry, SMS, caring contact, suicide prevention

## Abstract

**Introduction:**

Caring contact SMS text messages were designed as a brief suicide prevention intervention to support service users (*tangata whaiora*) after suicidal behaviour. The aim of this exploratory research was to evaluate responses to receiving a series of caring contact messages via SMS, culturally tailored to a New Zealand context.

**Method:**

Participants presenting to an Emergency Department after suicidal behaviour were sent a series of seven SMS text messages. In this qualitative study, participants were interviewed by phone to evaluate their experiences of receiving SMS. Individual interviews were audio-recorded and transcribed. Reflexive thematic analysis was performed in three cycles of coding.

**Results:**

Three themes were identified: connection to the caring essence of text messages, strengthening of self-agency by the message series, and cultural dimensions that support healing, including use of te reo Māori (Māori language).

**Conclusion:**

SMS is acceptable as a means to reach and connect with people after an episode of suicidal behaviour. Tailoring messages to an individual’s culture and context can potentially enhance their therapeutic value. Further research is recommended to evaluate upscaling through automated delivery, the use of apps, and use of language via SMS.

Caring contacts are brief, caring messages^1–3^ that express concern for a person^4^ and awareness of the person’s context.^5^ Caring contacts via short message service (SMS) are one means of providing support after a suicidal crisis. They are non-demanding, in that the person is not required to respond^6^ and they may facilitate communication with services.^7^ SMS is associated with reduced suicidal ideation, hospitalisation, and presentations to Emergency Departments^8^ and can be used during gaps in treatment^9^ to develop connection^10^ and coping skills.^11,12^ SMS has been found to be accessible, acceptable, inexpensive, asynchronous, and potentially scalable as an intervention.^11,13–15^

Emergency Departments are underutilised sites for suicide prevention.^4^ Brief interventions^16^ can reduce subsequent suicide attempts^17^ and increase linkages to care^18,19^ for service users and their support system during transitions such as after discharge from an Emergency Department (ED) or inpatient unit.^18,20,21^

New Zealand has a high national rate of suicide.^22^ In 2020, there were 612 confirmed suicide deaths with an age-standardised rate of 11.5 (95% CI: 10.6–12.4) deaths per 100,000 population.^23^ The suicide rate for Māori males was 25.5 per 100,000 Māori male population, 1.7 times that of non-Māori males. Each year 20,000 people attempt suicide and 150,000 have thoughts of ending their life.^24^ In 2023, more than 600 individuals presented to our ED with suicidal behaviour (suicide attempt, self-harm, or suicidal ideation), 14% (*n* = 86) had documented suicidal thoughts only, while 79% (*n* = 494) had attempted suicide or engaged in self-harm. Most were discharged to primary care and a smaller proportion to community mental health services.

In this study, we drew on person centred theory which emphasises the quality of the therapeutic relationship,^25^ self-determination theory which proposes autonomy, competence and relatedness in motivation,^26^ and mentalisation,^27^ referred to as theory of mind, the ability to understand the mind of another.^28^ We aimed to evaluate individuals’ experiences of receiving caring contacts after a suicide attempt, to explore engagement with a series of text messages and advance principles of a brief suicide prevention intervention. Indigenous perspectives of suicide are shaped by relationship to land, ancestors, and spirituality^29^ and understanding one’s role within a collective.^30^ The research team considered Māori health dimensions of health in designing the study.^29,31^ We preferentially use the term *tangata whaiora* rather than service user (Table 1).

**Table 1.** Glossary of Māori terms

Kupu/word	Definition
Kaimahi	Professionals – workers
Mana	One’s own authority, essence, or power
Mana whenua	Territorial rights, power from the land, authority over land or territory, jurisdiction over land or territory, power associated with possession and occupation of tribal land
Mātauranga Māori	Māori knowledge – the body of knowledge originating from Māori ancestors, including the Māori world view and perspectives, Māori creativity and cultural practices
Mauri	Life force, spirit
Tangata whaiora	A person seeking wellness
Tauiwi	Person coming from afar – a term used for those who are not Māori, but live and are part of Aotearoa New Zealand
Te reo Māori	The Māori language, indigenous to Aotearoa New Zealand
Te ao Māori	Māori worldviews
Te Tiriti o Waitangi	The constitutional document of Aotearoa New Zealand
Tikanga	Māori procedure, customs, lore, or rules
Tino rangatiratanga	Translated here as ‘self-determination’, it is the fullest expression of rangatiratanga – autonomy, sovereignty, control, or power
Whānau ora	Whānau (Wider family) wellbeing
Tapu	Sanctity; sacrality; sacred
Noa	To be free from the extensions of tapu, ordinary, unrestricted, void
Tikanga	Customary practices and protocols
Wairua	Spirit, soul – spirit of a person which exists beyond death
Whakawhanaungatanga	Process of establishing relationships and relating well to others
Whakapapa	Genealogy, lineage

## Method

This study has been reported according to standards for reporting qualitative research.^32^ The research was based in Auckland, an ethnically diverse city of 1.5 million people. The study was conducted at Health New Zealand Counties Manukau, an Emergency Department serving a catchment area of 537,000 (2018 census). The research team have backgrounds in academia, psychiatry and psychology, policy, quality improvement, suicide prevention education, and Māori perspectives of mental health.

Interpretive description methodology combined the research team’s clinical and research lens’ to practically apply findings to the field of practice.^33,34^ We collaborated with a clinical psychologist, peer specialists, and Māori and Pacific cultural specialists to design a series of text messages (Table 2). Peer specialists (people with lived experience of mental distress) evaluated the SMS design^35^ and tangata whaiora were invited to receive and evaluate the text messages.

**Table 2.** SMS series received by participants after attempted suicide

SMS series	Text content
Text 1 (Within Week 1)	Tēnā Koe [name], It’s [name] here from [location]. I was just wondering how you were going after yesterday. Remember for support freephone or text 1737 Arohanui (warm regards) [name]
Text 2 (Week 1)	Tēnā Koe [name], [name] here from [location]. Just seeing how you are doing? You are most welcome to freephone or text 1737 if you want to kōrero (talk) about how things are going. Take care. [name]
Text 3 (Week 2)	Kia ora [name], We were thinking of you. Hope you are feeling uplifted in your Mauri (energy) today. If you like to you can contact 1737 by free phone or text. Ngā mihi (Regards) [name]
Text 4 (Week 4)	Kia ora [name], Hope all is well with you and your whānau (family). You can Freephone or text 1737 at any time if you want a kōrero (chat). Ngā mihi (regards) [name]
Text 5 (Week 7)	Kia ora [name] [name] here. Thinking of you and wishing you well. For support or a kōrero (talk) Freephone or text 1737.
Text 6 (Week 11)	Tēnā Koe [name], Hope you are feeling settled in your wairua (spirits). Freephone or text 1737 for support if you want. Ngā mihi [name]
Text 7 (Week 12)	Kia ora [name], Hope everything is going well for you. Support or a kōrero (chat) continues to be here for you. Freephone or text 1737. Arohanui (warm regards) [name]
Optional on birthday	Happy Birthday [name], We hope it’s been ka pai and that this year brings you good things! From [name]

Two designated staff based in the ED on-call mental health team invited and verbally consented nine participants to receive a series of text messages over a 6-month timeframe. Adults (age 18–65) who presented to ED following a suicide attempt were included. Excluded were people with cognitive impairment or acute psychotic disorders requiring admission to an inpatient unit. Participants were given a resource card (Table 3). Phones and phone credit were made available to ensure equitable access. SMS were sent within 3 days, at 1 week and 2, 3, 4, 6, and 12 weeks of the ED presentation. Three months later, separate consent was gained from participants to evaluate the SMS series via phone. None of the research team had a role in participants’ care or assessment. A semi-structured interview guide was used. Interviews were audio-recorded and professionally transcribed. Field notes were made by the interviewer, indicating length and qualitative aspects of the interviews.

**Table 3.** Resource card given to participants in the Emergency Department

Karakia	Kia Piki Ake Te Mauri (Uplift the mauri)
Manawa mai te mauri nuku	Catch up with a friend or whānau
Manawa mai te mauri rangi	Spend time in te Taiao (nature)
Ko te mauri kei au, he mauri tipua	Eat good kai
Ka pakaru mai te pō	Sing or listen to waiata
Tau mai te mauri	Go for a walk, dance or kapa haka
Haumi e, hui e, taiki e!	Breathe deeply
Embrace the life force of the earth Embrace the life force of the sky The life force I have gathered is powerful And shatters all darkness Come great life force Join it, gather it, it is done!	
**Tautoko (Support)**	**Ngā Rauemi (Resources)**
Need to talk 1737	Manawa safety plan app
Lifeline 0800543354	Manaaki Ora app
Samaritans 0800 726666	The HAAAA app
Youthline 0800376633	Whānau M3 app
Tautoko Suicide Crisis Helpline 0508828865	Aunty Dee app
Depression Helpline 0800111757	Mental Health Foundation Website
Kia Ora	
Thanks for agreeing to receive some supportive text messages over the next 3 months. We are inviting people seen in the emergency department after a suicide attempt to receive these messages and this will be in addition to the support you may already receive. With your consent we will write to your GP to inform them you are receiving this support	
We hope these messages will be helpful but if you wish to stop receiving them please text reply STOP. Unfortunately, these text messages are not monitored but you can kōrero with someone by Freephone 1737 and if it is an emergency please dial 111	
At a later date, we would like to invite you to tell us what you think of the text messages. This feedback is completely voluntary and part of a research project based at the University of Auckland	

Transcripts were read for familiarisation and manually coded by all members of the research team. An inductive approach was used, guided by principles of reflexive thematic analysis.^36^ The first round of coding was conducted individually. An audit trail was documented containing memoranda, questions, and reflections. In the second round of coding, the transcripts were reviewed by an independent researcher. In the third round, coders conceptualised themes which were further refined. There was specific coding of Māori data (anonymised) to ensure the regard of *mātauranga Māori* (Māori knowledge) in the analytic process.

## Results

There were seven participants aged between 18 and 42 years, who identified as Cook Island Māori, European, Niuean, Indian, Korean, Māori, and Thai. There were three salient themes: connection to caring essence; self-agency strengthened by messages; and drawing on culture to support healing.

### Connection with caring essence embedded in SMS

‘A lot of this is about creating some sort of connection, that you maybe can feel that somebody is caring for you’. (Participant 4)

Participants reported feeling held in mind with kindness and compassion, and connection to the essence of the text messages conveyed in language and tone. They did not generally recall specific message content, rather the use of non-directive, personalised, and non-clinical language. The text messages were described as subtle, kind, warm, open, and reassuring. Some referred to contact as a way of being seen by the health system.

### *Self-agency* *strengthened by messages*

‘Not too intrusive, not too much, not going all in and trying to fix something…a lot of the time people just need someone there’. (Participant 1)

This theme emphasised participants’ motivation to determine how and when to engage with the messages, exercising agency in having control over interacting with messages, allowing them to engage at their own pace and according to their personal needs. Text messages were perceived as non-intrusive and not trying to fix anything. They reported feeling supported, not being pressured to reply, and appreciated prompts to access support. Messages were viewed as tangible entities on their phone that they could refer back to. Participants understood messages were not reciprocal and that they were not expected to reply.

### Drawing on culture to support healing

‘It’s a magical feeling when you find someone that speaks the same language as you, belongs to the same cultural background and understands the situation you’re in’. (Participant 5)

Participants expressed clear preferences for tailoring to their culture and circumstances, to be greeted and made to feel welcome in their own language. Some participants perceived some words to have quintessential healing or *rongoā* (Māori healing, translated as remedy). Certain Māori words such as *mauri* (life force) and *wairua* (spirit) were resonant. Māori language encompassed holistic aspects of health and spirituality, uplifting *mauri, mana* (one’s own authority, essence, or power), and vital qualities. Participants accepted the use of te reo Māori as a way of establishing relationships and connecting with non-physical, spiritual dimensions, and aspects of collective culture: *‘[Te Reo] Māori is part of New Zealand, there should be that option’*. Some tauiwi (non-Māori) participants found the use of indigenous language helped them feel they belonged: *‘It made me feel like I was at home in New Zealand’*.

## Discussion

The aim of this exploratory study was to evaluate participants’ experiences of receiving and engaging with a SMS brief suicide prevention intervention. Participants connected with the caring essence of messages and there was strengthened agency. In our context, healing was promoted by personalisation to culture. Our findings are in line with studies that demonstrate SMS as accessible and feasible to use in populations at risk of suicide and self-harm.^13,12,15^ A unidimensional SMS text message may not create relational depth,^37^ yet notions of care conveyed in messages hold elements of therapeutic value. Participants perceived they were held in mind, potentially enhancing reflective functioning.

Feeling understood and cared for^27^ is linked to self-agency in owning and making decisions to contact support. Caring contacts via SMS may endorse positive psychological growth, enabling a shift towards congruence and self-acceptance.^26^ This alteration in attitudes reduces barriers to care as an invitation to seek help is accepted.^8,15^ Messages can be further personalised at time points such as birthdays.^6^

SMS automation can provide a platform for psychoeducation and self-efficacy.^38^ The content of our messages was tailored to a New Zealand context, using te reo Māori. Young Māori are disproportionately represented in suicide deaths compared to non-Māori.^39^ In te ao Māori, individuals are part of a network of interconnected social relationships based on entities of *whakapapa* (genealogy), *tikanga* (indigenous Māori customs), *wairua* (spirit), *tapu* (sacredness, sanctity), *noa* (be free of tapu, unrestricted), *mauri* (spirit), and *mana* (authority, essence, or prestige). The messages were co-designed with these concepts in mind. Language is one way to bridge te ao Māori (the Māori world) and a collectivist system of healing in SMS messages by enhancing connection, mauri *(spirit)*, and whakawhanaungatanga *(establishing rapport)*.

Te reo Māori has been highlighted as a remedy in recovery.^35^ As we enter a language journey, it enables deepened understanding of tikanga and mātauranga Māori through a language lens. Te reo Māori is explicitly linked to mātauranga Māori and provides opportunities to explore certain cultural approaches that open pathways for inner reflection through uniquely Māori concepts.^40^ Feeling connected to indigenous language may engender a sense of belonging and enhance mental health and wellbeing.^41,42^ Language, culture, and identity are closely connected.^43^ Whilst some participants would have liked to have received messages in their own language, non-Māori participants responded positively to the cultural references in the messages which may reflect increasing familiarity with and acceptance and support of Māori language and culture.^44^

## Implications for practice and directions for future research

We note difficulties attaining ethics approval and recruitment for this pilot study, concerns related to approaching participants after suicidal behaviour, maintaining privacy of health records and the two-step consent required to receive the text messages and subsequently take part in the study. Research on this sensitive topic is challenging to design and conduct in an ED, with constraints on time, resources, and personnel. Most reported studies of caring contacts have recruited people following an ED presentation with suicidal behaviour following discharge from a psychiatric inpatient unit. However, many people with suicidal behaviour do not present to the ED nor require an inpatient admission. We recommend future research to explore whether caring contacts benefit people who disclose recent suicidal behaviour in less acute settings such as community mental health or primary care and the effects of caring contacts on people with suicidal thoughts who have not acted on them.

The main limitation of this study is the small sample size, which reflects the sensitive nature of this research.^12,45^ Malterud et al^46^ note data saturation to be closely tied to a specific methodology and advise caution in its adoption as a generic quality marker.^46^ We prefer the concept of information power^46^ which provides alternative guidance as to the adequacy of sample size when using qualitative methodology: relating to the study aim, sample, theoretical framework, dialogue, and analytic strategy. In this study, the purposive sampling, albeit small, reveals new experiences of receiving SMS that are relevant to the study’s aim to understand the utility of sending culturally tailored text messages to participants following an ED contact related to suicidal behaviours. Our dual clinician-researcher lens in using interpretive description methodology contributed to valuable rapport-building and communication during data collection and lent a strong reflexive quality to the analysis. Therefore, we cautiously suggest that SMS automation or caring contact messaging via a mobile application has potential benefits. Automation facilitates scalability, as an adjunct to treatment as usual or as the only service support after suicidal behaviour. More research is needed to determine if our identified themes are consistent with upscaling. Further evaluation of SMS for youth, males, and family members and the use of te reo Māori as a means of enhancing connection would be of value. Strengths of this study are text messages designed by people with lived experience of psychological distress and suicidal behaviour, having the same person send text messages and facilitate interviews and independent co-coding in addition to that conducted by the research team. The utilisation of mātauranga Māori (knowledge bases) and te reo Māori (language) in this research enhances the applicability and contextualisation for indigenous peoples internationally. We did not enquire into more detailed aspects of participants’ histories such as first-time versus previous suicide attempts and characteristics that may have inclined them to opt into receiving the SMS series.

## Conclusion

SMS is acceptable as a means to reach and connect with people who have attempted suicide. Text messages that are tailored to an individual’s culture and context may enhance their therapeutic value. Further research is recommended to evaluate upscaling through automated delivery, the use of apps, and acceptable use of language via SMS (Supplemental Material 1).

## Supplemental Material


Supplemental material - Caring contact SMS text messages following suicidal behaviour: Qualitative study
Supplemental material for Caring contact SMS text messages following suicidal behaviour: Qualitative study by Lillian Ng, Danielle Diamond, Denisse Sanchez and Mike Ang in Australasian Psychiatry

## Data Availability

Raw data is stored in accordance with New Zealand Health, and Disability Ethics Committees guidelines are not publicly available to preserve individuals’ privacy. The dataset supporting the conclusions of this article is available on reasonable request from the corresponding author.*
