# Supplementary material for: Caring contact SMS text messages following suicidal behaviour
Source: Australas Psychiatry. 2025 Oct 7;34(2):122–8. doi: 10.1177/10398562251382455 (PMC13062447; doi:10.1177/10398562251382455)
Supplement: Supplemental material - Caring contact SMS text messages following suicidal behaviour: Qualitative study [file sj-pdf-1-apy-10.1177_10398562251382455.pdf]

1. What do you think of the idea of receiving text-based messages?
2. What was it like to receive the messages? How did you feel about getting them?
3. What feedback do you have about the content, language and tone of the messages?
   Content – what did you think of what they said?
   Language – what did you think of the language used?
   Tone – how did they feel, were they pitched about right?
4. How can the texts be improved?
5. What is your feedback on the timing and frequency of these messages?
   What are your thoughts on the frequency?
   What time is optimal to receive them?
6. Were the texts helpful? In what way was receiving text message support following a suicide attempt helpful to you?
7. What specific messages were most helpful? Why?
8. Are there any downsides of receiving text message support?
9. How can we best tailor the messages to take into account your culture? How might we be more considerate of needs of Māori service users?
   How can the texts be more considerate of your culture?

Supplementary Table: Semi-structured interview schedule
